# DHX9 helicase is involved in preventing genomic instability induced by alternatively structured DNA in human cells

**DOI:** 10.1093/nar/gkt804

**Published:** 2013-09-17

**Authors:** Aklank Jain, Albino Bacolla, Imee M. del Mundo, Junhua Zhao, Guliang Wang, Karen M. Vasquez

**Affiliations:** Division of Pharmacology and Toxicology, College of Pharmacy, The University of Texas at Austin, Dell Pediatric Research Institute, 1400 Barbara Jordan Blvd. Austin, TX 78723, USA

## Abstract

Sequences that have the capacity to adopt alternative (i.e. non-B) DNA structures in the human genome have been implicated in stimulating genomic instability. Previously, we found that a naturally occurring intra-molecular triplex (H-DNA) caused genetic instability in mammals largely in the form of DNA double-strand breaks. Thus, it is of interest to determine the mechanism(s) involved in processing H-DNA. Recently, we demonstrated that human DHX9 helicase preferentially unwinds inter-molecular triplex DNA *in vitro*. Herein, we used a mutation-reporter system containing H-DNA to examine the relevance of DHX9 activity on naturally occurring H-DNA structures in human cells. We found that H-DNA significantly increased mutagenesis in small-interfering siRNA-treated, DHX9-depleted cells, affecting mostly deletions. Moreover, DHX9 associated with H-DNA in the context of supercoiled plasmids. To further investigate the role of DHX9 in the recognition/processing of H-DNA, we performed binding assays *in vitro* and chromatin immunoprecipitation assays in U2OS cells. DHX9 recognized H-DNA, as evidenced by its binding to the H-DNA structure and enrichment at the H-DNA region compared with a control region in human cells. These composite data implicate DHX9 in processing H-DNA structures *in vivo* and support its role in the overall maintenance of genomic stability at sites of alternatively structured DNA.

## INTRODUCTION

The integrity of genomic DNA is challenged by various insults within the cell, including base modifications by exogenous and endogenous sources, unscheduled cleavage at non-standard recognition sequences (1), strand breaks and expansions and deletions induced by non-canonical (i.e. non-B) DNA structures that can form transiently during replication and transcription (2–6). Most repetitive DNA motifs, including short tandem repeats (microsatellites), inverted repeats, alternating purine-pyrimidine tracts, runs of purines•pyrimidines (Pu•Py) and G-rich sequences, have the capacity to adopt alternatively structured DNA (non-B DNA structures), including slipped and looped-out conformations, cruciforms, left-handed Z-DNA, triplex (H-DNA) and quadruplex structures (7,8).

A number of studies aimed at addressing the consequences of non-B DNA in model systems (i.e. bacteria, yeast, mammalian cells and mice) concur with the conclusion that these conformations induce DNA damage responses and genomic instabilities, such as gross rearrangements and point mutations (3,9–11). Thus, the formation of non-B DNA has been proposed to underlie a number of human inherited diseases, including triplet repeat expansion diseases (12), recurrent chromosomal translocations (13), copy number variations (14) and gene conversion events (15).

Intra-molecular triplex DNA (H-DNA) forms at poly(Pu•Py) tracts with mirror repeat symmetry, where unpairing of the duplex in half of the tract allows one of the single strands to pair with the purine-rich strand of the remaining half of the duplex, thus forming a three-stranded helix (16). Genome-wide bioinformatics analyses indicate that regions with the potential to form H-DNA occur at an average of 1/50 000 bp in the human genome (17), and that these tracts are generally over-represented in promoter regions of genes and in the introns of genes involved in cell signaling and cell communication (18). H-DNA-forming sequences have been shown to block replication *in vitro* (6,19) and to promote DNA double-strand breaks (DSBs) in mammalian systems (9). Indeed, Pu•Py tracts have been found to co-localize with breakpoint hotspots in disease-related genes, such as the *c-MYC* gene in Burkitt lymphomas (20) and the *BCL2* gene in follicular lymphomas (21).

Previously, we demonstrated that the H-DNA-forming sequence from the human *c-MYC* gene promoter is mutagenic and induces large-scale deletions in mammalian cells (2). Most deletion breakpoints were found to occur near the H-DNA sequence and were characterized by microhomologies, implicating microhomology-mediated end-joining in their processing. Subsequently, we reported that the same *c-MYC* H-DNA-forming sequence induced genetic instability (predominantly in the form of deletions and translocations) in a transgenic mouse model (22). Thus, these results suggest that triplex-induced DSBs may underlie a number of recurrent translocations in diseases such as Burkitt’s lymphomas in human (20) and t(12;15) BALB/c plasmacytomas in mice (23).

Despite correlations between the formation of triplex DNA and genetic instability, the proteins that recognize and process these structures and the mechanisms that generate mutations at these sites are still poorly understood. Several DNA repair proteins have been shown to recognize triplex DNA both in eukaryotes and prokaryotes, including mismatch repair and nucleotide excision repair proteins (24–27). The recognition of non-B DNA by repair proteins may either trigger site-specific mutagenesis through error-generating repair, or prevent instabilities, depending on a number of factors including the types of underlying non-B DNA structures (triplexes, hairpins/loops, etc.) involved (28,29).

DNA helicases, such as the RecQ family of helicases defective in the Werner (WRN) and Bloom syndromes, and the Fanconi anemia group J protein (FANCJ), have been shown to resolve triplex DNA structures (30,31) and other secondary conformations *in vitro* (32). The Bloom, WRN and Fanconi Anemia syndromes are characterized by genomic instability and increased susceptibility to cancers (33,34). Consistent with the helicase activities *in vitro*, genetic assays have revealed that both the RecQ-type, and other helicases such as Pif1, serve to counteract non-B DNA-dependent instability, both in vertebrates (human and worm) (35,36) and invertebrates (bacteria and yeast) (37,38).

In search of components of the cellular machinery that process and resolve triplex DNA structures, we recently isolated DHX9 as part of a protein complex that binds triplex DNA in human embryonic 293 T kidney cells (39). DHX9 belongs to the DEXH family of superfamily 2 helicases (40), performing critical functions during transcription, translation and repair (41–43). Homozygous knockout leads to embryonic lethality in mice (44). *In vitro*, the DHX9 helicase domain has been shown to be active on DNA, RNA and DNA/RNA hybrid substrates (45). However, we found that purified DHX9 exhibits a preferred helicase activity on inter-molecular DNA triplexes, as compared with duplex and forked DNA substrates (39).

Herein, we addressed the role of DHX9 in processing naturally occurring H-DNA structures *in vivo*. Specifically, we used a *supF* reporter plasmid-based system containing the human *c-MYC* H-DNA-forming sequence in transfection experiments conducted in wild-type and siRNA-treated (DHX9-depleted) osteosarcoma U2OS cells. We found that DHX9 recognizes H-DNA in the context of supercoiled plasmids, thereby protecting the structure from mung bean nuclease (MBN) cleavage, and that siRNA-mediated DHX9 depletion increased the frequencies of mutations induced by H-DNA, with deletions being mostly affected. Chromatin immunoprecipitation (ChIP) assays revealed an enrichment of DHX9 at H-DNA structures relative to canonical B-DNA, and electrophoretic mobility shift assays (EMSA) confirmed the specific association between DHX9 and H-DNA *in vitro*. Hence, we conclude that DHX9 aids in preserving genomic stability by resolving mutagenic H-DNA structures in human U2OS osteosarcoma cells.

## MATERIALS AND METHODS

### Plasmid substrates

**Figure 1.**
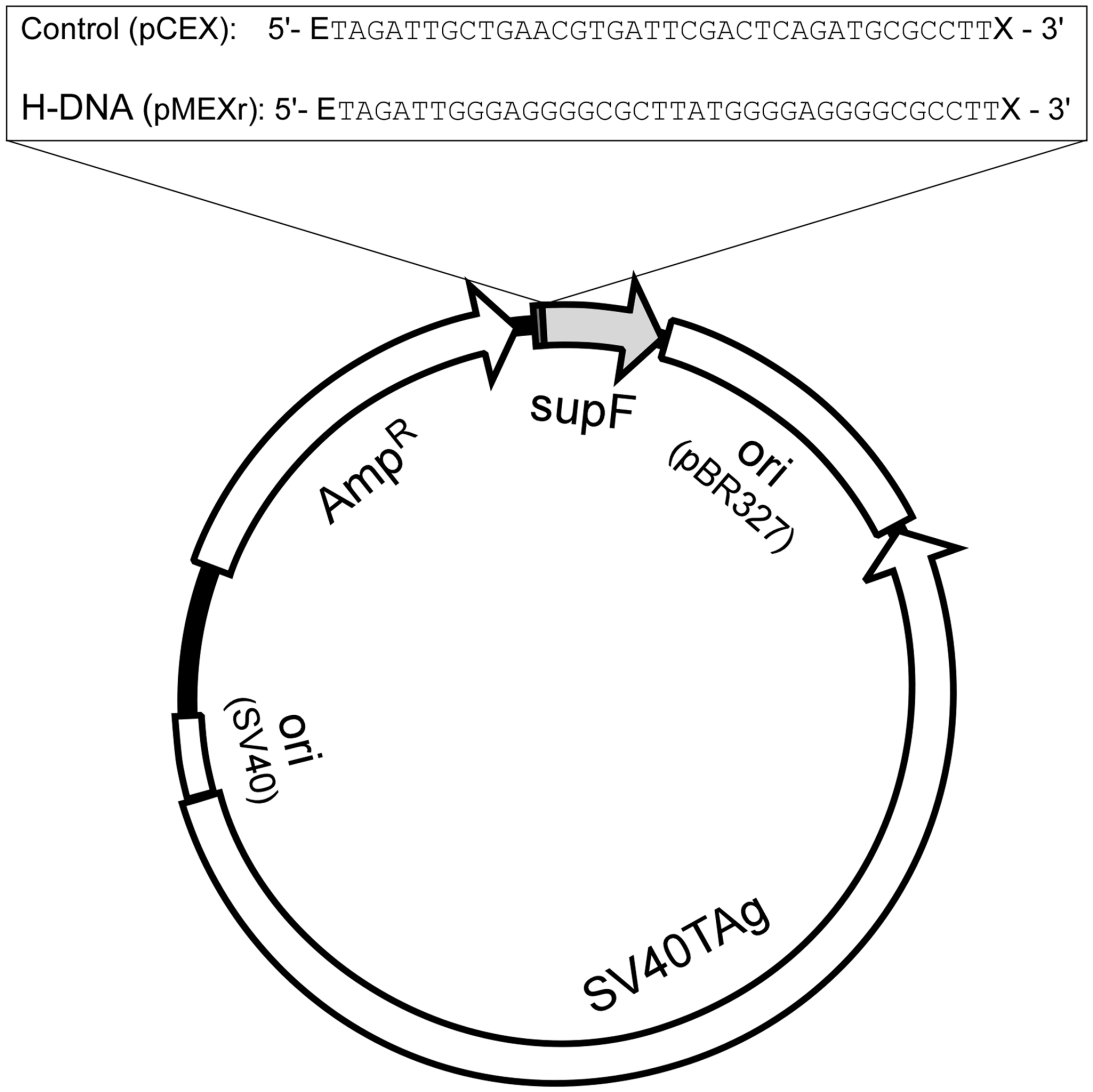
Plasmid features. Schematic of the pSP189 shuttle vector map showing the location of the control (pCEX) and H-DNA-forming (pMEXr) inserts cloned at the EcoRI (E) and XhoI (X) sites 4 bp upstream of the *supF* gene.

Sequences from the human *c-MYC* gene promoter capable of forming H-DNA structures, and control sequences that form canonical B-DNA were cloned into the shuttle vector pSP189 at the EcoRI and XhoI sites, as described (2,36). pSP189, which contains the *supF* mutation-reporter gene, the ampicillin resistance gene, the pBR327 replication origin and the SV40 viral replication origin, was used to conduct blue/white screening of mutant plasmids generated in human osteosarcoma U2OS cells. The resulting pMEXr and pCEX plasmids (Figure 1), containing the *c-MYC* H-DNA-forming sequence or control sequence, respectively, were purified by CsCl gradient ultracentrifugation. The final constructs were verified by restriction analyses and direct sequencing using primers hybridizing within the ampicillin resistance gene.

### Cell culture and siRNA treatment

Human osteosarcoma U2OS cells were cultured in Dulbecco’s modified Eagle’s media supplemented with 10% fetal bovine serum. Transfections with siRNA were performed twice, at 24 h and 48 h, with 100 nM siRNA targeting either the DHX9 helicase (Gene ID 1660) or the control luciferase mRNAs in six-well plates using Lipofectamine 2000 (Invitrogen, Carlsbad, CA, USA), as per manufacturer’s instructions. The sequences for the siRNA oligonucleotides were 5′-guaaaugaacguaugcuga-3′ for DHX9 and 5′-cttacgctgagtcttcga-3′ for the luciferase mRNAs (Thermo Scientific Research, Inc. South Logan, UT, USA). Seventy-two hours after the first transfection, cells were harvested for immunoblotting to assess the level of gene knock-down. For immunoblotting, whole-cell lysates were prepared following the NucBuster protein extraction kit (EMD Bioscience, San Diego, CA, USA) protocol. A total of 30 µg of protein was resolved by SDS–PAGE and immunoblotted on polyvinylidene flouride (PVDF) membranes (Bio-Rad, Hercules, CA, USA). Primary antibodies used for western blotting were rabbit anti-DHX9 (Abcam, Inc. Cambridge, MA, USA) and rabbit anti-β-actin (Abcam, Inc.), which are known to react with the relevant human proteins. Signals were detected by the ECL Western Blotting kit (GE Healthcare, Buckinghamshire, UK).

### Expression and purification of recombinant DHX9 protein

A recombinant DHX9 bacmid (46) (a generous gift from Prof. Frank Grosse, Jena, Germany) was produced according to the manufacturer (Bac-to-Bac Baculovirus expression system, Invitrogen) instructions. Briefly, donor plasmid pFastBAC-His-DHX9 was transformed into competent DH10Bac *Escherichia coli* cells, and, subsequently, PCR was used to verify the directionality of the *DHX9* cDNA in the recombinant bacmid. A forward pUC/M13 primer and a reverse primer from the internal *DHX9* cDNA (5′-TCCATGTTCTCGTGCAAAAATCCT-3′) were used for the PCR reaction. Recombinant DHX9 bacmid was transfected into Sf9 insect cells and cultured in sf 900 III serum-free media (GIBCO) in the presence of Cellfectin (Life Technologies, Inc., Invitrogen). Supernatants were collected after 72 h and DHX9 protein expression was verified by western blot using an anti-DHX9 antibody (B-9, sc137232, Santa Cruz Biotechnology, Inc.). Virus stocks were prepared with a multiplicity of infection of 0.01–0.1. Baculovirus stocks containing the human DHX9 vectors were used to infect 1–2.5 × 10^8^ Sf9 cells in 15 culture dishes (150 mm in diameter) at a multiplicity of infection of 1–5. Cells were harvested 72 h post-infection and washed with ice-cold phosphate-buffered saline. Whole-cell extracts were prepared at 4°C by homogenizing cells in 5 ml of lysis buffer [25 mM HEPES-KOH (pH 7.9), 150 mM NaCl, 5 mM MgCl_2_, 1 mM DTT, 10% glycerol, 1x proteinase inhibitor and 0.01% NP40]. Three different types of chromatography beads were used to purify the DHX9 protein, which were pre-equilibrated in base buffer [25 mM HEPES-KOH (pH 7.9), 5 mM MgCl_2_, 1 mM dithiothreitol, 10% glycerol, 1x proteinase inhibitor and 0.005% NP40]. Cell extracts were first centrifuged at 13 000 rpm for 15 min, and supernatants were mixed with 1 ml of Affi-Gel Blue Gel (100–200 mesh, Bio-Rad) and rotated at 4°C for 2.5 h. Recombinant DHX9-bound Affi-Gel Blue Gel was then loaded onto a Poly-Prep chromatography column (Bio-Rad), and beads were washed with 300, 600 and 750 mM KCl in 6 ml of base buffer. The DHX9 protein was eluted from the Affi-Gel Blue Gel with 1 M KCl in 3 ml of base buffer. The eluted protein was diluted with base buffer (3 volumes), mixed with 5 ml of P11 beads, rotated at 4°C for 2.5 h and centrifuged for 3 min at 1000 rpm at 4°C. Supernatant was diluted with 1 volume 1 mM imidazole in base buffer supplemented with 400 mM KCl. The protein solution was subsequently mixed with 0.5 ml of HIS-Select Nickel Affinity Gel (Sigma) and rotated overnight at 4°C. Beads were washed twice with 6 ml of base buffer containing 15 mM imidazole and 400 mM KCl, and once with 3 ml of base buffer containing 15 mM imidazole and 200 mM KCl. Washing consisted of rotating for 5 min and centrifuging for 2 min at 6000 rpm. Finally, DHX9 protein bound to HIS-Select Nickel Affinity Gel was mixed with elution buffer [25 mM HEPES (pH 7.9), 15% glycerol, 0.005% NP40, 100 mM KCl, 1 mM DTT, 150 mM imidazole, 5 mM MgCl_2_], rotated for 20 min at 4°C and supernatant was stored at −70°C. This procedure yielded >90% pure recombinant DHX9, as assessed by SYPRO tangerine staining.

### Helicase assays

For helicase assays, 4 μg of plasmid DNA was incubated with 30 nM DHX9 protein in 24 µl of reaction buffer [10 mM Tris–HCl (pH 7.5), 5 mM DTT, 100 µg/ml BSA, 5 mM MgCl_2_, 5 mM ATP or 5 mM APM-PNP and 10% glycerol] at 35°C for 30 min. AMP-PNP (adenosine 5′-(β,γ-imido)triphosphate, Roche, Nutley, NJ, USA), is a non-hydrolyzable ATP analog that competitively inhibits ATP-dependent enzyme systems. Next, 10 units (1 µl) of MBN (New England Biolabs, Beverly, MA, USA), which cleaves at single-stranded and triplex-duplex DNA junctions, were added for 20 min at 35°C. Reactions were terminated by the addition of 10 mM EDTA, quick chilling on ice and loading buffer (40% sucrose, 0.1% bromophenol blue and 0.1% xylene cyanol). Reaction products were resolved on 1% agarose gels in 1x TAE buffer at room temperature, and bands were visualized by ethidium bromide staining and quantified using the ImageJ software (http://rsbweb.nih.gov/ij/). The percentage unwinding by DHX9 was determined by calculating the net amount of open circular (OC) plus linear (L) DNA divided by the total amount of DNA [i.e. closed circular (CC) + L + OC] that remained at the end of the reactions. To map the MBN cleavage sites at the H-DNA region and their protection by DHX9, 4 µg supercoiled plasmid was preincubated with 30 nM DHX9 for 30 min at 35°C in 24 µl of reactions in the presence of 5 mM ATP, as before. Ten units MBN was added, and incubation continued for 20 min. Reactions were brought to 100 µl, extracted with phenol/chloroform and then ethanol precipitated. DNA was labeled with 10 units T4 DNA polymerase (New England Biolabs) in a 18 µl of reaction volume containing the manufacturer recommended buffer at 12°C for 7 min. In the absence of dNTPs, this protocol activated the endonuclease activity of the polymerase as to degrade ∼1.5–2 kb of DNA from any DSBs induced by MBN. Two microliters of solution containing 1 mM each of dATP/dGTP/dTTP and dCTP-[α−^32^P] (6000 mCi/mmol, PerkinElmer, Waltham, MA, USA) was added, and DNA synthesis was allowed to proceed at 12°C for 30 min. After ethanol precipitation, plasmid DNA was cleaved with Eco0109I and AhdI (New England Biolabs, Beverly, MA, USA), further purified by additional ethanol precipitations and electrophoresed on 1% agarose gels. The gels were first stained with ethidium bromide, dried and then exposed to a PhosphorImager (Typhoon FLA 9000, http://www.ge.com) for visualization.

### ChIP

The *in vivo* recognition of H-DNA-forming regions by the DHX9 helicase was determined by ChIP assays using the Simple ChIP Enzymatic Chromatin IP kit (Cell Signaling Inc., Santa Cruz, CA, USA), as described (24), with minor modifications. In brief, U2OS cells were grown to 60–70% confluence in Dulbecco’s modified Eagle’s media, and ∼5 µg of pMEXr or pCEX plasmids was transfected using the GenePORTER transfection reagent. After 48 h, U2OS cells were harvested and subjected to cross-linking with 1% formaldehyde, followed by quenching with 125 mM glycine for 5 min. The chromatin lysates were treated with micrococcal nuclease and sonicated on a water bath-based sonicator (Epigentek group Inc., USA) to achieve average DNA fragment lengths of ∼500 bp. Immunoprecipitation was performed using rabbit monoclonal antibodies against human DHX9 (Abcam, Inc. Cambridge, MA, USA) and rabbit immunoglobulin G (IgG) (Cell Signaling Inc., Santa Cruz, CA, USA). After immunoprecipitation, PCR was performed using 2x GoTaq Green Master Mix (Promega Inc., Madison, WI, USA) for 26 cycles consisting of 30 s at 95°C, 30 s at 56°C and 45 s at 72°C, and a final 5 min extension time at 72°C, on a Bio-Rad thermal cycler. The primers used were: forward primer (Fp), 5′-gcccccctgacgagcatcac-3′; reverse primer (Rp), 5′-tagttaccggataaggcgcag-3′. Amplified PCR products were resolved by electrophoresis through 1.5% agarose gels and visualized using a Bio-Rad ChemiDoc Imaging system. Band intensities were assessed using the ImageJ software. The percentage immunoprecipitation was defined as the enrichment of immunoprecipitated DNA relative to the amount of input DNA based on the quantification of the respective PCR products. ChIP experiments were repeated a minimum of three times.

### EMSA

To investigate the binding of DHX9 to intra-molecular triplex structures *in vitro*, 100 nM purified DHX9 was incubated with 100 nM 5′-radiolabeled double-stranded DNA substrate or a pre-annealed 5′-radiolabeled intra-molecular triplex substrate containing a 3′-overhang in a 25 µl of reaction mixture in binding buffer [20 mM Tris–HCl (pH 7.5), 50 mM NaCl, 1 mM DTT, 1 mM EDTA, 10 mM MgCl_2_, 5% glycerol, 5 mM AMP-PNP and 1 µl 1x protease inhibitor (Complete Mini EDTA-free, Roche)] for 30 min at 30°C. After incubation, samples were mixed with 6 µl of 5x Nucleic Acid Sample Loading Buffer (Bio-Rad) and then electrophoresed through 8% native polyacrylamide gels (29:1 acrylamide/bis-acrylamide, Bio-Rad) in 89 mM Tris-borate, 2 mM EDTA (TBE) at 175 V for 3.5–5 h. Gels were subsequently dried and exposed (24–72 h) to a Phosphor Screen for visualization of the radiolabeled substrates on a Typhoon FLA-9000 Biomolecular Imager and Image Quant TL v7 software (GE). Oligonucleotides used to form the double-stranded substrate (5′-ctt gag ctt gag ctc aag ctc aag-3′) and *c-MYC*-related intra-molecular triplex with a 3′-overhang (5′-ccc ctc cct ttt tgg gag ggg cgc tta tgg gga ggg cag tcg agc g-3′) were from The Midland Certified Reagent Company, Inc., Midland, TX, USA. The 5′-ends were labeled with [γ-^32^P]-ATP (Perkin-Elmer, Boston, MA, USA) using T4 polynucleotide kinase (New England Biolabs) for 60 min at 37°C. The unincorporated nucleotides were removed by size-exclusion chromatography using MicroSpin G-25 columns (GE, Buckinghamshire, UK). Annealing of the radiolabeled oligonucleotides was performed in 50 µl of reactions containing 50 mM NaCl, 1 mM EDTA, 10 mM MgCl_2_ and 20 mM Tris–HCl (pH 7.5), followed by heating to 95°C for 5 min and cooling for ∼12 h. Double-strand and intra-molecular triplex formation were confirmed by gel electrophoresis and circular dichroism (data not shown).

### Mutagenesis assays

To determine H-DNA-induced mutagenesis, 5 µg of *supF* mutation-reporter plasmids (pMEXr or pCEX) were transfected, along with a second round of siRNA treatment, in U2OS cells, using the GenePORTER transfection reagent (Gene Therapy System, Inc. San Diego, CA, USA). Forty-eight hours after plasmid/siRNA transfection, cells were collected and plasmid DNA was isolated using the QIAprep Spin Miniprep kit (QIAGEN Inc., Valencia, CA, USA) according to the manufacturer’s recommendations. After isolation, DNA was treated with Dpn1 restriction enzyme to remove any plasmids that did not replicate in the human U2OS cells, extracted with phenol/chloroform, ethanol-precipitated in 0.3 M sodium acetate (pH 5.2), 70% ethanol and 20 μg/ml glycogen at −20°C overnight, and dissolved in sterile H_2_O. Mutations in the *supF* gene were detected in the *E. coli* strain MBM7070, in which the *lacZ* gene is inactivated by an amber stop codon, using a blue/white screen on 5-bromo-4-chloro-3-indolyl-β-D-galactopyranoside, isopropyl-β-D-thiogalactoside and ampicillin plates. In the presence of su―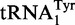, the product of the plasmid-encoded *supF* gene, amber suppression is relieved and bacterial colonies appear blue; however, most mutations in the *supF* gene yield an inactive su ―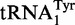, which produces white bacterial colonies (36). Mutation frequencies were calculated as the number of white (mutant) colonies divided by the total (blue + white) number of colonies. Experiments were repeated a minimum of three times. The Holm–Sidak one-way analysis of variance test was used to assess the statistical significance between groups. To determine the types of mutation events that occurred in the human cells, DNA was isolated from randomly selected white colonies, sequenced using primers that hybridized within the ampicillin resistance gene, and the results aligned to the respective wild-type sequences.

### Illumina DNA sequencing

Forty-five micrograms of plasmid DNA (pCEX and pMEXr) was transfected into 4 × 10^6^ U2OS cells using GenePORTER (GTS Inc., San Diego, CA, USA) and recovered 48 h post-transfection using the Hirt’s method (47). DNA was first cleaved with DpnI to fragment unreplicated plasmid DNA and then treated with 10 units lambda exonuclease for 3 h to fully degrade both the DpnI-cleaved fragments and any contaminating genomic DNA. DNA that was resistant to DpnI and exonuclease digestion, i.e. plasmids that replicated in U2OS cells, was subjected to next-generation sequencing on a HiSeq 2000 sequencing system (http://www.illumina.com) to detect base variants (mutations) that might be present in varying proportions in the entire plasmids. The less reliable sequence reads generated from the first and last 20 nucleotides of each read were removed, whereas the internal more accurate read sequences were aligned to the corresponding plasmid maps. Total base variants for the forward and reverse reads at each nucleotide position were normalized by their relative coverage. To attain a smooth distribution profile of mutations present along pCEX and pMEXr, the normalized variants were summed over 50 consecutive bp and plotted as 1-bp sliding windows aligned to the first nucleotide position of the plasmids.

## RESULTS

### DHX9 helicase recognizes H-DNA structures in plasmids

**Figure 2.**
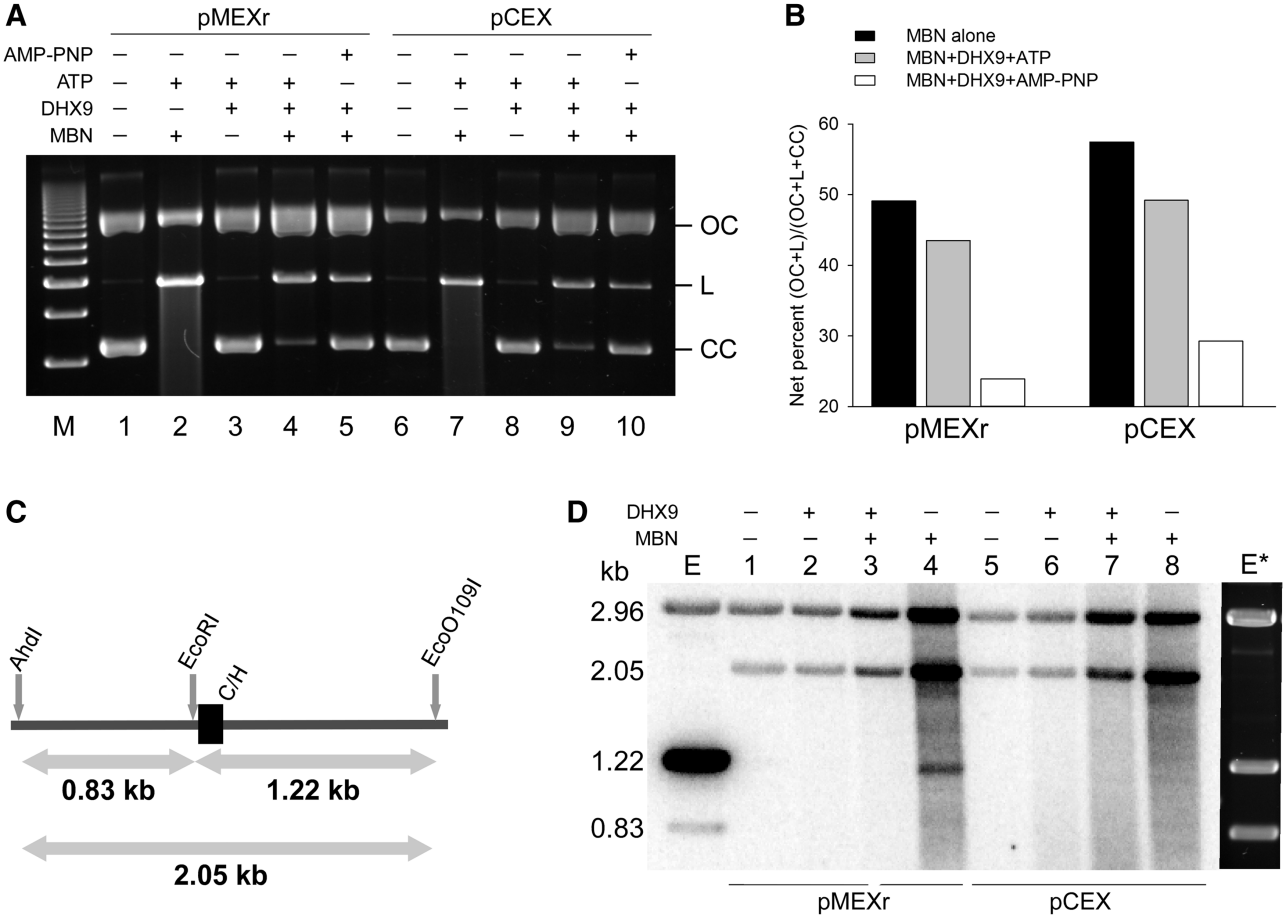
Human DHX9 recognizes DNA secondary structures in plasmids. (**A**), representative agarose gel of CC pMEXr (lanes 1–5) and pCEX (lanes 6–10) incubated with 30 nM purified recombinant human DHX9 protein (lanes 3–5 and 8–10) in the presence of 5 mM ATP (lanes 3 and 4, and 8 and 9) or 5 mM AMP-PNP (lanes 5 and 10) followed by MBN cleavage. Lane M, 1 kb DNA marker. (**B**), plot of net percentage of *OC* and linear (*L*) DNA released from total DNA (*OC + L + CC*) (*CC* = closed circular DNA) at the end of the reaction from two experiments, as described in Panel (A). The net percentage was defined as *F = f_s_ – f_c_*, were *f_s_* was the %[(*OC + L*)/(*OC + L + CC*)] for each sample, and *f_c_* was the average %[(*OC + L*)/(*OC + L + CC*)] for the two samples without MBN (i.e. lanes 1, 3 and 6, 8, respectively). (**C**)*,* schematic of the diagnostic restriction sites (AhdI and EcoO109I) used to map MBN-specific cleavage in pMEXr. Grey arrows, lengths of restriction fragments released from pCEX and pMEXr when cleaved by AhdI, EcoO109I and EcoRI, which are located several bp from the cloned inserts; C/H, position of the control (C) and H-DNA-forming (H) inserts in pCEX and pMEXr, respectively. (**D**), MBN cleavage mapping. PhosphorImager scan of an agarose gel after electrophoresis of pCEX (lanes 1–4) and pMEXr (lanes 5–8) pre-incubated with 30 nM DHX9 (lanes 2 and 3, and 6 and 7) in the presence of 5 mM ATP, treated with 40 units MBN (lanes 3 and 4, and 7 and 8), end-labeled with T4 DNA polymerase and cleaved with Eco0109I and AhdI. Lane E, control lane containing pMEXr cleaved with EcoRI, labeled with T4 DNA polymerase, and then cleaved with AhdI and EcoO109I; E*, ethidium bromide staining of lane E.

We first determined whether DHX9 had the capacity to recognize H-DNA structures in plasmids because, unlike synthetic inter-molecular triplex DNA, its formation requires negative supercoiling and unwinding of duplex DNA. We conducted these studies *in vitro* using purified recombinant human DHX9 and MBN, which cleaves plasmid DNA at single-stranded regions, triplex-duplex junctions and nicks (48). MBN alone increased the amounts of nicked and linear molecules relative to the starting materials by ∼50% for the H-DNA-containing plasmid, pMEXr (Figures 1, 2A lanes 1–3 and B), and by ∼60% for the control plasmid, pCEX (Figures 1, 2A lanes 6–8 and B). Thus, both plasmids contained sequences that were substrates for MBN, which likely included short regions of denatured AT-rich sequences, non-B conformations (cruciforms from short inverted repeats and H-DNA) and nicks. Pre-incubation with DHX9 helicase (30 nM) alone did not alter the gross electrophoretic migration pattern (Figure 2A, lanes 3 and 8). However, pre-incubation with DHX9 followed by MBN treatment caused a 5–10% reduction in substrate available to MBN cleavage in the presence of ATP, and a stronger (∼30%) reduction in the presence of AMP-PNP, a non-hydrolyzable analog of ATP, both in pMEXr and pCEX (Figure 2A, lanes 4 and 5, and 9 and 10 and Figure 2B, bars 2 and 3, and 5 and 6). We conclude that DHX9 inhibited MBN cleavage by competing for binding to the sites also recognized by MBN (i.e. alternative DNA structures containing single-stranded regions and nicked DNA); AMP-PNP might have served to immobilize DHX9 at such sites, effectively precluding access by MBN.

To assess whether the H-DNA-forming insert was one of the sites of MBN cleavage, pCEX and pMEXr were treated with MBN and then cleaved with Eco0109I and AhdI, which yielded two plasmid fragments: a 2.96 kb fragment containing the vector sequences and a 2.05 kb fragment containing the control (pCEX) or H-DNA-forming (pMEXr) inserts. The inserts are adjacent to an EcoRI recognition site and, thus, further cleavage with EcoRI splits the 2.05 kb fragment into 0.83 kb and 1.22 kb (Figure 2C). The diagnostic fragments of 0.83 and 1.22 kb were detected following labeling of EcoRI-cleaved plasmids with T4 DNA polymerase and subsequent cleavage with AhdI and EcoO109I. Labeling of the longer fragment was consistently more robust than that of the smaller fragment, both in pCEX and pMEXr (Figure 2D, lanes E and E*). On MBN treatment under conditions where most supercoiled molecules were cleaved (Figure 2A) and plasmid degradation began to appear, two radioactively labeled fragments of ∼0.83 and ∼1.22 kb in size were revealed, which were specific to pMEXr (Figure 2D, compare lanes 4 and 8). From this and other gels, the size of the fragments was consistent with MBN cleavage ∼20–30 nt downstream to the EcoRI site, toward the 3′ half of the H-DNA-forming insert. Thus, the H-DNA-containing insert formed secondary DNA structures in a subpopulation of pMEXr molecules *in vitro*, most likely H-DNA structures, which were recognized and cleaved by MBN. Pre-incubation of the samples with DHX9 and ATP before MBN treatment precluded visualization of the 0.83 and 1.22 kb bands (Figure 2D, lanes 3 and 7), supporting the view that the helicase provided strong protection against MBN cleavage by binding the DNA secondary structure(s) formed by the *c-MYC* gene promoter insert. In summary, we conclude that DHX9 recognized, and possibly resolved, H-DNA structures formed in supercoiled plasmid DNA.

### H-DNA structures are recognized by DHX9 *in vivo* and *in vitro*

**Figure 3.**
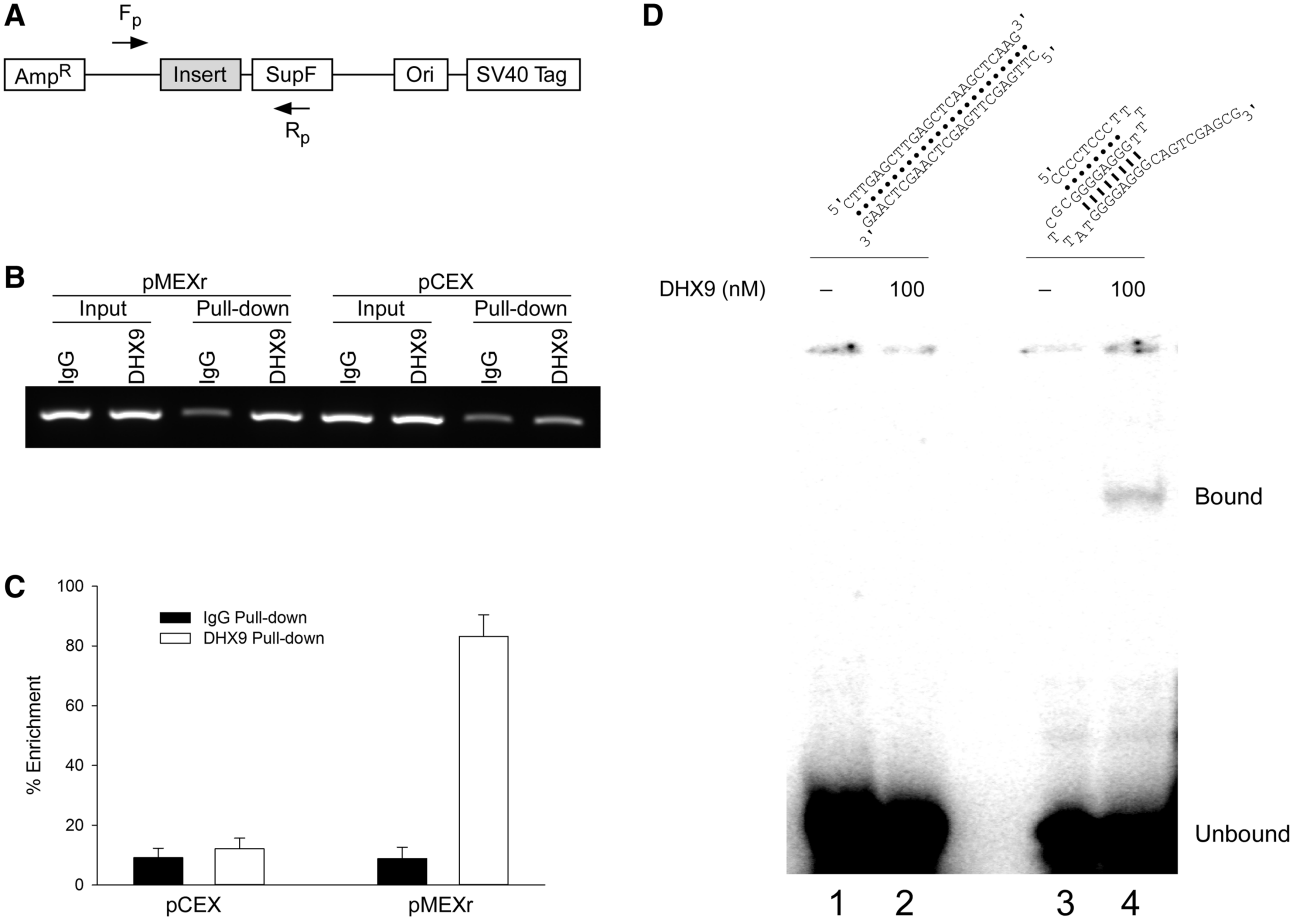
Human DHX9 recognizes H-DNA-forming sequences in U2OS cells and *in vitro*. (**A**), schematic of the plasmids and the primer pairs used for PCR amplification in ChIP assays. *F_p_* and *R_p_*, forward and reverse primers, respectively. (**B**), representative agarose gel image of PCR products from the ChIP assays. (**C**), plot of the percentage enrichment relative to input DNA from three independent ChIP assays, as shown in (B). (**D**), *top*, cartoon of predicted duplex (*left*) and intra-molecular triplex (*right*) DNA structures formed by the oligonucleotides. We note that because the oligonucleotide used for the duplex DNA substrate is self-complementary, both intra-molecular hairpin structures and inter-molecular duplex DNA may be formed by the oligonucleotide; *bottom*, EMSA of DHX9 binding to the *c-MYC* sequence-related triplex structure (100 nM) or duplex DNA (100 nM) in the presence of 5 mM AMP-PNP. Lane 1, duplex DNA only; lane 2, duplex DNA and 100 nM DHX9; lane 3, triplex DNA only; lane 4, triplex DNA and 100 nM DHX9.

To further characterize the role of DHX9 in processing H-DNA structures, we performed ChIP assays to determine the extent to which DHX9 interacted with the H-DNA region in human cells. The H-DNA structure-forming (pMEXr) and control (pCEX) plasmids were transfected into U2OS cells; 48 h later, cells were harvested and chromatin was immunoprecipitated using monoclonal antibodies against human DHX9 and control IgG proteins. Input and immunoprecipitated samples were amplified by PCR with primers flanking the inserts (Figure 3A). The DHX9 antibody pulled down significantly (∼6-fold) more DNA when the pMEXr plasmid was used, compared with pCEX (Figure 3B and C). As anticipated, the amounts of total DNA (input DNA) from cell lysates were similar in wild-type cells for pCEX and pMEXr and chromatin pull-down by IgG antibodies only yielded background signals, as assessed by PCR analyses (Figure 3B). These data provide direct evidence for the enrichment of DHX9 at the *c-MYC* H-DNA-forming region compared with that of control B-DNA *in vivo*, suggesting a specific protein–DNA interaction in cells.

To confirm that DHX9 was able to bind the *c-MYC* H-DNA structure(s) formed by pMEXr, we performed EMSA on a synthetic *c-MYC* sequence-containing oligonucleotide pre-folded into a specific intra-molecular triplex structure, as assessed by circular dichroism, containing a 3′ overhang as depicted in Figure 3D (top). A mobility-shifted (bound) species was revealed in the presence of 100 nM DHX9 and AMP-PNP (Figure 3D, lane 4), which was lacking in the absence of protein (Figure 3D, lane 3). Replacement of AMP-PNP with ATP resulted in a 33% reduction in the bound species, suggestive of helicase activity, and no mobility shift was observed when the reaction mixture contained a 100-fold (i.e. 10 µM) excess of non-radioactive H-DNA competitor (data not shown). With the duplex DNA substrate, no mobility-shift was apparent (Figure 3D, lanes 1 and 2) in the presence or absence of DHX9. These data confirm the ability of DHX9 to recognize H-DNA structure(s) formed by the *c-MYC* insert of pMEXr.

### DHX9-depletion increases H-DNA-induced mutations in human cells

**Figure 4.**
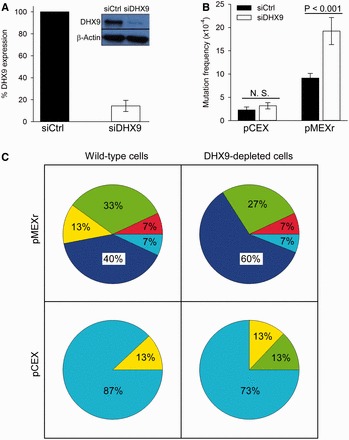
Mutation frequencies and spectra affecting *supF* gene function in wild-type and DHX9-depleted human U2OS cells. (**A**), representative western blot of DHX9 in U2OS cells treated with siRNA and plot of percentage DHX9 expression (average of three experiments). (**B**), frequencies of *supF* gene mutations for pCEX (control) and pMEXr (H-DNA) transfected into human U2OS cells treated with siRNA. *N.S.*, not significant. (**C**), Pie charts displaying the mutation spectra of pCEX and pMEXr transfected into wild-type and DHX9-depleted human U2OS cells; 15 random mutant colonies per group were analyzed; percentages were rounded. Dark blue, large (>100 bp) deletions; green, medium size (50–100 bp) deletions; yellow, small (<50 bp) deletions; red, insertions; light blue, point mutations.

Having established that DHX9 can recognize H-DNA structures in plasmids, we asked whether DHX9 is involved in reducing DNA structure-dependent genetic instabilities by resolving these mutagenic secondary structures *in vivo*. Thus, we determined the spontaneous and H-DNA-induced mutation frequencies in wild-type and DHX9-depleted human osteosarcoma U2OS cells. To achieve DHX9-depletion, we transfected the cells with siRNAs targeting either the DHX9 or a control luciferase mRNA. Treatment with 100 nM DHX9-specific siRNA oligonucleotides decreased the DHX9 protein levels by up to 90% relative to control siRNA, as assessed by western blotting (Figure 4A). To determine mutation frequencies, the *supF* mutation-reporter plasmids (pMEXr or pCEX) were transfected into wild-type or DHX9-depleted U2OS cells, and rescued and screened for mutations 48 h later. In control siRNA-treated cells, pMEXr caused an ∼4-fold increase (from 2.4 ± 0.6 × 10^−^^4^ to 9.2 ± 0.1 × 10^−^^4^) in mutation frequencies relative to pCEX (Figure 4B), as anticipated (2). These values were ∼10-fold greater than the background mutation frequencies arising from the MBM7070 bacterial population (3.5 × 10^−^^5^ for pCEX of 201 747 colonies and 5.0 × 10^−^^5^ for pMEXr of 199 507 colonies), and similar to prior determinations obtained using the same reporter system (36). By contrast, DHX9-depleted cells showed a more prominent (6-fold) increase (from 3.2 ± 0.7 × 10^−^^4^ to 19.3 ± 2.9 × 10^−^^4^) in mutations for pMEXr compared with the control pCEX (Figure 4B). Thus, whereas there was no difference in spontaneous mutation frequencies between the wild-type and DHX9-depleted cells for the control pCEX plasmid, DHX9 depletion caused a significant (*P* < 0.001) 2-fold increase in H-DNA-induced mutations in pMEXr (Figure 4B). Thus, we conclude that DHX9 played a significant role in suppressing H-DNA-induced mutations in human cells.

### Deep sequencing reveals a similar mutation landscape along the plasmid backbones

**Figure 5.**
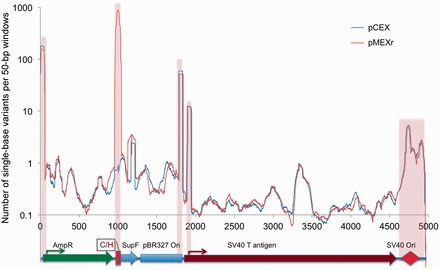
Spontaneous mutations in plasmid DNA. Landscape of single base substitutions present in the pCEX and pMEXr population following replication in human U2OS cells. Plasmid DNA was recovered from U2OS cells and single base mutants along the entire plasmid were detected by Illumina next-generation sequencing (see ‘Materials and Methods’ section). *y-*axis, total number of base variants in sliding 50-bp windows; *x*-axis, position along the plasmid map superimposed to plasmid genetic features. Pink highlight, selected regions with high numbers of variants.

The results reported earlier in the text raised the question as to whether negative supercoiling might have promoted mutation hotspots along the plasmid backbone in the control plasmid pCEX, which were repressed and shifted toward the H-DNA-forming region in pMEXr. If this were the case, then DHX9 could be regarded as a rather non-specific effector of DNA repair. To address this issue, we transfected pCEX and pMEXr into U2OS cells, selectively isolated the plasmid population that underwent DNA replication in the human cell line 48 h after transfection, and then subjected this DNA to deep sequencing (average coverage, 7946x for pCEX and 7326x for pMEXr). The landscape of single base variants summed over 50 bp sliding windows exhibited five peaks representing mutation hotspots (Figure 5), four of which were common to both pCEX and pMEXr. These four areas mapped to the promoter of the *Amp* gene, the pBR327 bacterial replication origin, the promoter region of the SV40 T antigen gene and the SV40 replication origin, all regions known to be active elements for the generation of mutations in these plasmids (2,3). The fifth peak, which was specific to pMEXr and also displayed the highest number of base variants (∼1000) along the plasmid landscapes, coincided with nt range 960–1040, which corresponds to the location of the H-DNA-forming sequence (nt 1022–1044 and Figure 1). Close examination of the areas abutting the H-DNA-forming insert also revealed a modest increase in mutations in pMEXr compared with pCEX. For example, in the nt range 1040–1111, encompassing the *supF* gene, there were 78 base variants in pCEX and 404 in pMEXr, a ∼5-fold increase, which might reflect the greater mutation frequencies observed in pMEXr relative to pCEX (Figure 4). Thus, apart from the H-DNA-forming region and its immediate flanking areas, no obvious sections were present that would indicate a shift in mutation hotspots from the H-DNA-forming region to other loci along the plasmid backbone of the control pCEX plasmid. We conclude that, at least within the confines of this reporter system, the decrease in mutation frequencies observed in the presence of DHX9 may reflect specific interactions between the helicase and DNA secondary structures formed by the H-DNA-forming region of the human *c-MYC* gene.

### DHX9-depletion has a moderate effect on H-DNA-induced deletion spectra

To compare the types of mutation induced by either H-DNA or the control plasmid between DHX9-depleted and wild-type cells, we randomly selected 15 mutant clones per group from three different experiments and characterized the mutations by DNA sequencing. For pMEXr in wild-type U2OS cells, 87% mutations were deletions (Figure 4C), as expected (2). Of these, 40% consisted of large-scale deletions (>100 bp in length), 33% of medium-size deletions (50–100 bp in length) and 13% of small deletions (<50 bp in length) (Figure 4C). The remaining ∼13% mutations were equally divided (7% each) between short insertions and point mutations (Figure 4C). A slightly greater fraction of pMEXr plasmids underwent large-scale deletions in DHX9-depleted cells (60% compared with 40% in wild-type cells) (Figure 4C).

For pCEX, spontaneous mutations included single-base substitutions for the most part (87% in wild-type and 73% in DHX9-depleted cells), with the remaining 13 and 27%, respectively, consisting of deletions (∼13% comprising medium-size deletions in DHX9-depleted cells, Figure 4C—note that these percentages were not rounded to sum up to exactly 100%). The majority (∼70%) of point mutations were found across the entire *supF* mutation-reporter gene. These results indicate that large deletions were unlikely to occur in the absence of H-DNA, even in a DHX9-depleted background, whereas they were induced by H-DNA.

**Figure 6.**
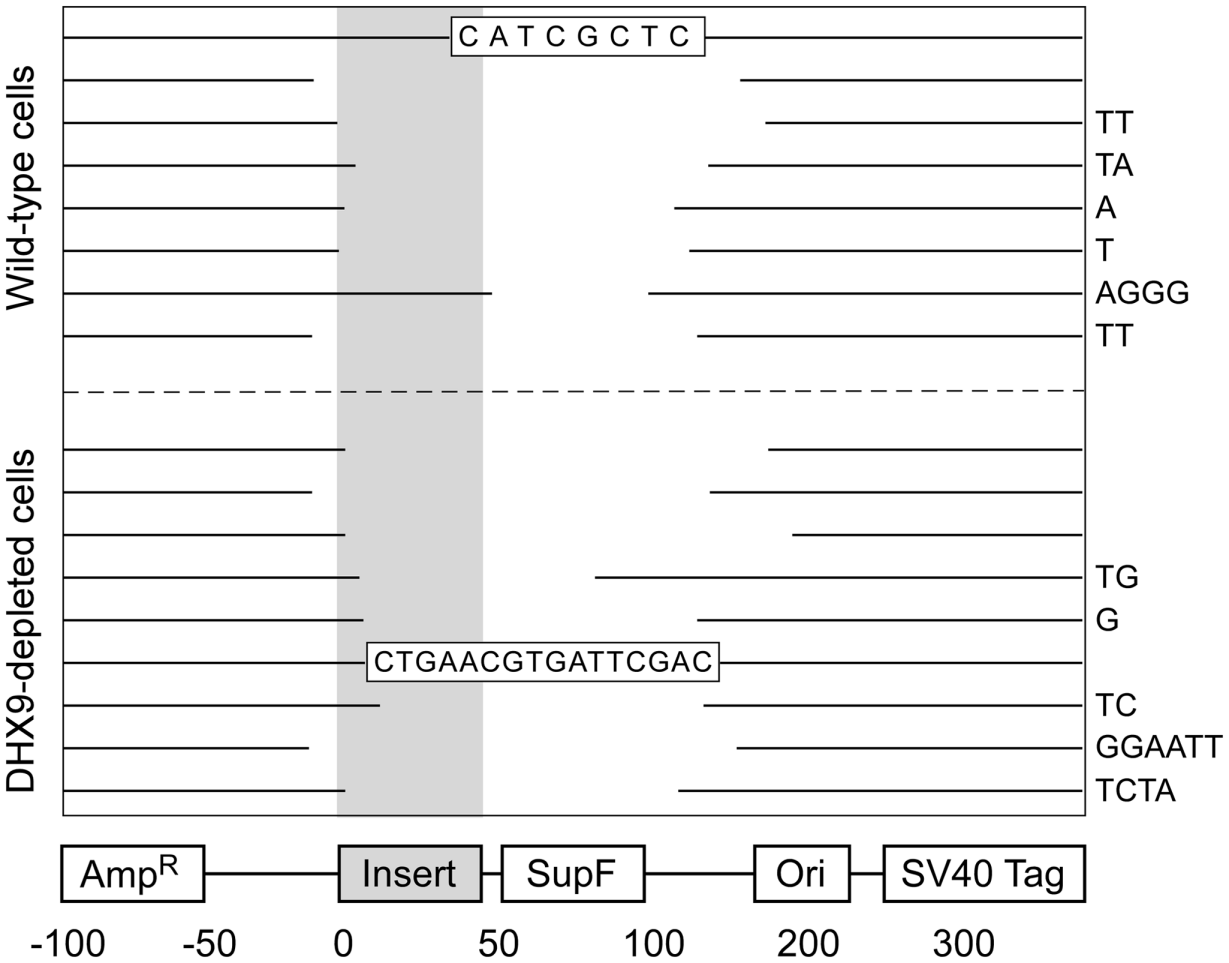
Representation of deletion mutants. Gaps represent the deleted sequences aligned to the plasmid map (*bottom*) for 17 of the deletion mutants shown in Figure 3. Right-hand side nucleotides, micro-homologies at the junctions; shaded bar, triplex-forming region in pMEXr; boxed sequences, insertions.

The breakpoint junctions of 17 medium- and large-scale deletions (nine from DHX9-depleted cells and eight from wild-type U2OS cells) included 11 cases (∼60%) where the entire *c-MYC* H-DNA-forming insert was deleted, five cases where the proximal breakpoint occurred within the insert, and one case where both the proximal and distal breakpoints were located 3′ of the insert (Figure 6). In three clones, the proximal breakpoint occurred within 5 bp from the vector/insert boundary, a critical region of helical distortion at the transition between B- and H-DNA, expected to lead to DNA strand breaks (9). Two deletion events were accompanied by a short *de novo* insertion (16 and 8 bp long); 11/17 clones were characterized by the presence of short (1-6) bp microhomologies, a characteristic of the non-homologous end-joining (NHEJ) pathway, whereas in the remaining five clones, no homologies were observed. We did not observe any significant difference in the extent of microhomologies at the joined ends between the wild-type and DHX9-depleted cells. These data support the conclusion that DHX9 reduced H-DNA-induced instability, in part by offering protection to the broken DNA ends leading to deletions. However, it did not alter to a significant extent the mechanics of end-fusion.

## DISCUSSION

The main findings of this study may be summarized as follows. First, the H-DNA-forming sequence from the human *c-MYC* gene promoter conferred sensitivity of supercoiled plasmid DNA to cleavage by MBN, and pre-incubation with human DHX9 helicase afforded protection from MBN cleavage. We interpret these data to indicate that H-DNA structures at the purine•pyrimidine-rich *c-MYC* gene sequence are induced by negative supercoiling (49) and cleaved by MBN (48). DHX9 binds and possibly resolves these H-DNA structures through its helicase activity, thereby preventing MBN cleavage.

Second, the *c-MYC* sequence-containing plasmid (pMEXr) increased the frequencies of mutations in human osteosarcoma U2OS cells, and transient DHX9 deficiency exacerbated these mutations, which comprised of deletions for the most part. Otherwise, the *c-MYC* sequence did not appear to alter the location and occurrence of mutation hotspots along the plasmid backbones, which were indistinguishable between pCEX and pMEXr. Most deletion breakpoints were characterized by short (1–6 bp) microhomologies, suggesting a microhomology-mediated end-joining pathway as the predominant underlying repair mechanism. These data support the conclusion that DHX9 plays a significant role in counteracting H-DNA-induced genetic instabilities in U2OS cells.

Third, DHX9 co-immunoprecipitated with the *c-MYC* promoter sequence in wild-type U2OS cells and bound to a bona fide triplex structure formed by the same *c-MYC* sequence *in vitro*, implying the occurrence of specific protein–DNA recognition. From these composite results, we propose that DHX9, either alone or as part of a complex, recognizes H-DNA conformations *in vivo*, conferring partial protection from the ensuing structure-dependent genetic instabilities.

One confounding factor in the interpretation of our study is the potential occurrence of DNA strand breaks at the time of transfection in human (and transformation in bacterial) cells, particularly at sites of alternative DNA structures, which may be present on the purified supercoiled molecules. We addressed the kinetics of DSBs induced by an H-DNA-forming plasmid following plasmid transfection in a previous study (2) and found that breaks were induced relative to a control plasmid both upstream and downstream (within ∼50 bp) of the H-DNA region within a 48 h period. Thus, whereas early breaks may have been consequent to transfection, late breaks were most likely generated after the initiation of plasmid DNA replication, supporting the notion that non-B DNA serves as an intrinsic cellular effector of DNA instability. The unwinding of H-DNA by DHX9 demonstrated here is consistent with our previous studies *in vitro*, which showed that recombinant DHX9 unwound synthetic inter-molecular triplex DNA substrates. The helicase activity was shown to proceed in a 3′→5′ direction in an ATP-dependent fashion and required a short single-stranded 3′ overhang (39). In this study, we confirm these conclusions using a *c-MYC* sequence-specific single-stranded oligonucleotide that folded onto itself to form an intra-molecular triplex containing a 3′-overhang. These kinds of overhang are absent in the types of intra-molecular triplexes formed on supercoiled pMEXr, raising the question as to the requirement for DHX9 loading on naturally occurring intra-molecular triplexes. On both pCEX and pMEXr, the amounts of supercoiled plasmid remaining after MBN treatment of plasmids pre-incubated with DHX9 were consistently greater in the presence of AMP-PNP than in the presence of ATP. Because nicks are obviously absent on supercoiled plasmids, these data suggest that DHX9 is capable of loading onto H-DNA substrates (and possibly other non-canonical DNA structures) in the absence of overhanging free ends on native supercoiled DNA. Thus, we suggest that in the presence of AMP-PNP, DHX9 remained stably bound at the H-DNA structure, effectively protecting it from MBN cleavage. In the presence of ATP, DHX9 translocation would resolve H-DNA structures, which however could re-form under the torsional stress of negative supercoiling. DHX9 has also been reported to unwind quadruplex structures formed by both DNA and RNA molecules, R- and D-loops and forked structures (50,51). In addition, the RecQ and FANCJ helicases have been shown to unwind non-B DNA conformations (30,31). Thus, our study expands the repertoire of enzymes that display helicase activity on DNA secondary structures *in vitro* and provides support for the existence of this type of activity within human cells.

Non-B DNA-forming sequences may induce genetic instabilities in the forms of deletions, translocations and single-base substitutions (3,8,13,22,36). Herein, we show that DHX9 is required to counteract these instabilities in human U2OS cells. Indeed, from the mutation frequency results, it appears that at least half of the H-DNA structure in the assay was attended to by DHX9, and not another helicase, suggesting some division of labor among structure-specific DNA-dependent helicases in the cell. Using the same plasmid system used herein, we previously showed that stable knock-down of the WRN helicase caused an increase in mutation frequencies with H-DNA- and Z-DNA-forming-sequences in U2OS cells. However, no selectivity was observed for any particular type of mutation (deletions versus single-base substitutions). We suggested that cellular adaptation to constitutive WRN knock-down, including a hyper-oxidative environment (36), in addition to WRN deficiency, was involved in the overall process. Herein, the use of transient knock-down may have prevented cellular adaptation to DHX9 deficiency (52,53), supporting the view that our current approach may have more directly revealed the effect of protein deficiency than stable knock-down, as issues with cellular adaptation were mitigated. In *Saccharomyces cerevisiae*, absence of the Pif1 DNA helicase was shown to cause extensive variations in DNA copy numbers of tandem repeat arrays of CEB1, a human minisatellite sequence with the ability to adopt quadruplex structures (38). Similarly, in *E. coli*, loss of the RecQ DNA helicase increased 8–10-fold the mutation frequencies of a triplex-forming sequence from the human *PKD1* gene in plasmids (37). Thus, evidence is mounting in support of the conclusion that several helicases evolved to recognize and resolve alternative DNA structures *in vivo*, and that their absence or insufficiency may cause a surge in genome instability.

**Figure 7.**
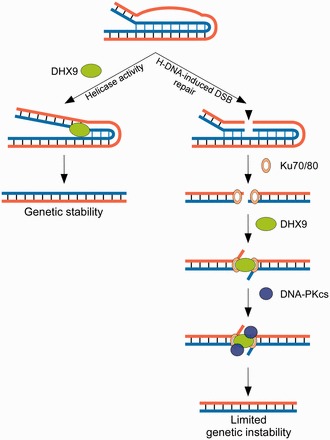
Model depicting the involvement of DHX9 in the processing of H-DNA (intra-molecular triplexes) structures. The left panel represents the first pathway in which the helicase activity of DHX9 resolves the mutagenic H-DNA structure, thus preserving genetic stability. The right panel represents the second pathway in which DHX9 participates in micro-homology mediated end-joining of H-DNA-induced DSBs perhaps by protecting the free ends, and therefore limiting genomic instability. These two models are not mutually exclusive.

The analyses of mutant clones revealed microhomologies at most breakpoints, consistent with a microhomology-mediated end-joining repair of non-B DNA-induced DSBs (2,3,54). NHEJ requires DNA-PKcs, Ku70/Ku80 heterodimer, XRCC4-DNA ligase IV (55,56) and topoisomerase II-alpha (57). DHX9 has been shown to form a complex *in vivo* with topoisomerase II-alpha in a type of interaction that requires the presence of non-coding RNA molecules and the ubiquitin-conjugating enzyme E2I (58). In addition, DHX9 has been reported to be phosphorylated by DNA-PKcs (59), to co-immunoprecipitate with Ku70 and BRCA1 (60,61) and to co-localize with BRCA1, γ-H2AX and Ku70 in nuclear foci in response to DSB-inducing agents (43,60). These observations suggest that both RecQ-type helicases and DHX9 are intimately involved in DNA repair during NHEJ. Our ChIP assays indicate that DHX9 was stably bound to the H-DNA-forming region of the *c-MYC* promoter sequence. Thus, it is likely that the ChIP assays captured specific interactions between DHX9 and H-DNA in chromatin-plasmid complexes in human cells. If the role of DHX9 was solely to unwind H-DNA, then we would expect this interaction to be transient and to require either a helicase-dead domain, as in the case of Pif1 binding to G4-forming sequences (62), or the use of AMP-PNP to be detected by ChIP. Hence, it is possible that DHX9 may play a dual function in U2OS cells, as outlined in Figure 7. On the one hand, the protein may recognize H-DNA structures and unwind them, thereby counteracting the occurrence of DSBs and ensuing instabilities, as shown herein. On the other hand, after H-DNA-induced DSBs have formed at or near H-DNA structures, DHX9 may participate in the subsequent NHEJ repair process, perhaps by unwinding the termini to facilitate access to the breaks by other proteins involved in DSB repair or by assisting in the search for microhomologies.

## FUNDING

National Institutes of Health [CA097175 and CA093729 to K.M.V.]. Funding for open access charge: NIH [CA097175 and CA093729].

*Conflict of interest statement*. None declared.
